# Assessment of water quality in moatize, mozambique: possible human health risks from coal mining and use

**DOI:** 10.1007/s10653-026-03013-1

**Published:** 2026-02-12

**Authors:** Micaela Arlete José Chapo Cossa, Hassina Mouri, Robert B. Finkelman, Vicente Albino Manjate, Kim Dowling

**Affiliations:** 1 Department of Geology, Faculty of Science, University of Johannesburg, Johannesburg, 2006 South Africa; 2 University of Texas at Dallas, Richardson, TX 75080 USA; 3 Ministry of Mineral Resources and Energy, National Institute of Mines, Maputo, Mozambique; 4 School of Science, STEM College, RMIT University, Melbourne, VIC 3001 Australia

**Keywords:** Coal, Potential-toxic-elements, Water-quality, Human-health-assessment, Moatize

## Abstract

This study evaluated harmful elements and associated health risks in Moatize, Mozambique’s surface and groundwater, aligning with SDGs 3 (good health) and 6 (clean water). During both wet and dry seasons, 30 water samples were collected. Ions (Na^+^, K^+^, Ca^2+^, Mg^2+^, SO_4_^2−^, HCO_3_^−^, NO_3_^−^, F^−^, and Cl^−^) were analyzed using Ion Chromatography (IC), while concentrations of PTEs (Ba, Co, Cr, Cu, Mn, Mo, Pb, Sr, V, U, and Zn) were determined in water, coal, and ash samples by ICP-MS. Results indicated that PTE levels in coal and coal ash exceeded global average trace element concentrations. Moatize’s water chemistry is mainly influenced by natural geological processes, especially rock weathering. Most water samples showed Pb and Se levels above the WHO (2021). Guidelines for drinking-water quality (4th ed., incorporating the first and second addenda). World Health Organization and Mozambican Water Quality Limits (MWQL). In surface water, Pb ranged from 0.006 to 0.11 mg/L during the dry season and 0.003–0.11 mg/L during the wet season; groundwater levels ranged from 0.003 to 0.016 mg/L, with two samples exceeding the WHO/MWQL limit of 0.01 mg/L. Se was elevated only in dry season groundwater (0.017–0.022 mg/L), exceeding the MWQL of 0.01 mg/L. The Pollution Index (PI) ranged from low (0) to highly polluted (392.77) due to PTEs. The most common pollutants were Pb > Se > Mn > Cu in dry season surface water and Pb > Se > Cu > Mn in wet season surface water and groundwater. Health risk assessments indicated potential non-carcinogenic issues from oral exposure (HQoral > 1), especially from NO_3_^−^, Cu, and Se, with children being more vulnerable. Conversely, dermal exposure (HQdermal < 1) did not pose significant health risks for any group.

**Supplementary Information:**

The online version contains supplementary material available at 10.1007/s10653-026-03013-1.

## Introduction

Coal mining has historically played a crucial role in the economic growth of nations. It remains a significant contributor to global energy sources, accounting for a substantial portion of worldwide fossil fuel use (Zhang et al., 2022). However, the extraction of coal via both open-pit and underground methods is inherently linked to significant environmental degradation and subsequent public health crises (Finkelman et al., 2021). These activities trigger substantial land degradation, atmospheric pollution, and water contamination, which, in turn, induce severe detrimental effects on biodiversity and human health. Coal deposits contain a range of potentially toxic elements (PTEs), including arsenic (As), cadmium (Cd), mercury (Hg), lead (Pb), selenium (Se), boron (B), fluorine (F), manganese (Mn), molybdenum (Mo), nickel (Ni), beryllium (Be), copper (Cu), thorium (Th), uranium (U), vanadium (V), zinc (Zn), barium (Ba), cobalt (Co), and antimony (Sb), which are released into the environment during mining and burning (Fu et al., 2018). These substances can cause environmental damage and human health problems, such as chronic respiratory diseases, heart conditions, and cancer (Siddiqui et al., 2022).

Coal is a complex substance containing far more than just carbon, including various potentially toxic elements, radioactive elements, silicates, carbonates, and organic compounds like polycyclic aromatic hydrocarbons (PAHs) (Sarkar et al., 2021). This diverse composition is known to cause severe water pollution throughout the coal lifecycle. The elemental composition of coal critically influences water quality and, consequently, human health. As water and air interact with coal, acid mine drainage (AMD) is generated as sulphide minerals present in coal are oxidized, producing sulphuric acid that mobilises and releases potentially toxic elements (PTEs) into the surrounding water environment (Muedi et al., 2025; Sarkar et al., 2021). Coal ash leaching from storage sites also releases concentrated PTEs and toxins into water, with spills causing immediate severe contamination. Direct runoff and spills from coal processing introduce harmful chemicals and particles into waterways (Wang et al., 2022). Additionally, thermal pollution from power plant cooling water reduces dissolved oxygen in aquatic ecosystems (Hasii & Gasii, 2024).

Both surface and underground coal mining significantly damage ecosystems and hydrological systems, drastically affecting global surface and groundwater quality, quantity, and characteristics (Mahato et al., 2018). Large-scale mining operations can alter water levels and flow in shallow aquifers (Ahmat et al., 2024), leading to contamination as polluted water infiltrates through blasting-induced fractures (Lu et al., 2024). Underground mining can change groundwater patterns, while surface mining often degrades surface waters through runoff (Finkelman et al., 2021).

Water tainted with PTEs poses substantial health hazards. Consuming this water, whether by drinking, cooking, or eating contaminated fish, can lead to serious health problems (Finkelman et al., 2021). These include skin lesions, neurological issues, various cancers, and cardiovascular disease. Such exposure can also impair child neurodevelopment and negatively affect adult kidney and reproductive health (Ahmad et al., 2021). Additionally, both children and adults may experience kidney dysfunction, bone fragility, lung damage, cancer, and fatigue as potential outcomes (Mustafa & Hassan, 2024).

Open-cast mining generates high concentrations of PTEs that persist because their artificial generation rapidly outpaces natural decay, causing long-term environmental harm by degrading water (Gautam et al., 2021), disrupting ecosystems, and interfering with plant processes (Motshumi et al., 2025). Critically, these PTEs can enter human bodies through the food chain, posing a serious threat to public health (Kumari & Bhattacharya, 2023).

Despite the growing recognition of these detrimental effects, coal remains a critical energy resource for both developing and developed economies, ensuring its continued utilization for the foreseeable future (Chugh et al., 2023). Coal mining is practiced in many parts of the world, including Europe, China, the USA, Australia, and in some African countries (Zhao et al., 2017). Currently, the environmental and health impacts resulting from coal mining are of great concern; however, the persistent demand for energy perpetuates its use, with numerous studies continuing to highlight this critical issue (e.g., Xiang et al., 2021; Zhang et al., 2023). In some African countries, coal is a key resource that is highly exploited due to its abundance, necessity for energy needs, and low extraction cost (Marove et al., 2022). According to Hancox (2016), coal is the primary source of energy in South Africa and Botswana, accounting for over 90% of electricity generation. However, few studies focus on the environmental impacts of coal mining in Africa. Adeniyi et al. (2022) found that in Nigeria, coal mining has impacted the environment with the contamination of arable land, surface and groundwater, and resulted in biodiversity losses linked to AMD generated from mining waste and tailings. Furthermore, Hassan (2023) investigated the effects of coal mining on environmental sustainability in South Africa. The results demonstrated that environmental degradation in South Africa is linked to coal mining, and the researchers called upon government and institutions to require greater environmental sustainability. In Moatize, studies consistently document the generation of AMD and the associated environmental risks stemming from coal mining operations. Pondja et al. (2017a) investigated the impacts of coal mine, with Acid Base accounting (ABA) results showing uncertainties in Net Neutralizing Potential (between − 20 and +20 kg CaCO_3_ t^−1^). They advised controlling elevated concentrations of sulfates, Mn, Ca, and Mg in mining water.

The Moatize coalfield is part of the vital Zambezi River system (including the Revúboè and Moatize Rivers), which is a crucial water source for domestic and agricultural purposes for the local community. A study by Marove et al. (2020) investigating the hazardous element leaching from coal and ash observed that Cr and Mn were enriched in slightly acidic leachates. Furthermore, As leached from high-arsenic ash exceeded the WHO permissible limits. Their findings also indicated that all water samples ranged from “uncontaminated” to “moderately contaminated” with hazardous metals. Also, Pondja et al. (2017b) reported that mining operations in Moatize regularly release untreated PTEs into the Zambezi River basin. Despite the findings by Marove et al. (2020) and Pondja et al. (2017b), the effects of mining operations on water quality and human health in the area were not addressed. Specifically, their research did not investigate the widespread occurrence of PTEs and their associated health effects resulting from the release of this untreated water. Globally, the extensive adverse effects that coal mining has on the local eco-system (Habib & Khan, 2021) combined with the reliance of the local population on water resources within the Moatize Coal Basin, Tete Province, Mozambique, this study therefore aimed to: (i) carry out a detailed geochemical analysis of a comprehensive set of chemical elements including Ba, Co, Cr, Cu, Mn, Mo, Pb, Sr, V, U and Zn in coal, coal ash, surface water, and groundwater, (ii) evaluate the potential linkages between the chemical composition of coal and ash and the observed PTEs concentrations in surface and groundwater, (iii) assess the contamination levels in surface and groundwater within the Moatize region, (iv) assess the potential non-carcinogenic health risks posed by the PTEs through ingestion and dermal exposure and (v) delineate the spatial distribution of PTEs across the study area. The epistemological value of this work is grounded in its contribution to the whole set of global studies on contamination associated with coal mining across Africa, addressing the critical need to assess environmental and human health impacts for sustainable development.

### Study area

**Fig. 1:**
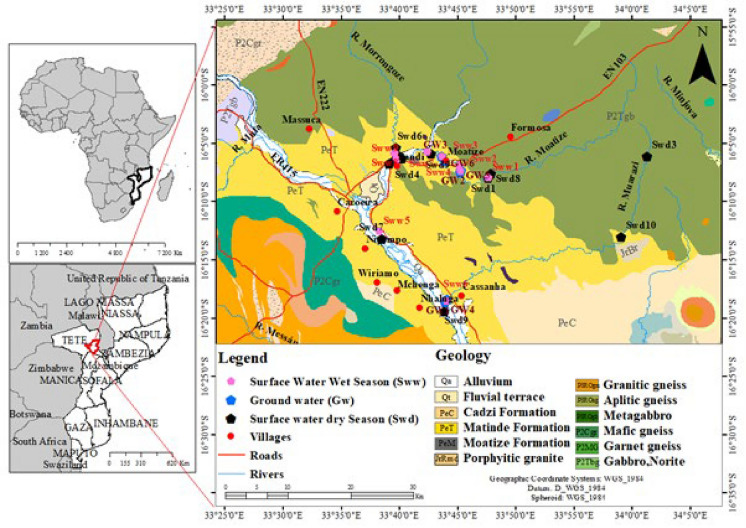
Location map showing the geology of the study area with sampling points of surface water and groundwater, roads, and villages (modified after Marove et al., 2022)

The Moatize District, Tete Province, is situated in central Mozambique, between latitudes 15°55′ 0″ S and 16°25′ 0″ S, and longitudes 33°25′ 0″ E and 34°05′ 0″ E (Fig. 1). Moatize exhibits a local steppe climate characterized by low annual rainfall. The average annual temperature is 26.5 °C, with maximum temperatures reaching 40 °C. The wet season (October–March) presents the highest precipitation, approximately 800 mm annually, and the dry season (April–September) exhibits the lowest precipitation, around 640 mm annually (MAE, 2014). The dominant vegetation in Moatize is influenced by both climate and soil type, featuring high and intermediate forests in the lower-lying areas along major river courses (Marques & Ferrara, 2007).

The Moatize Coal Basin is a graben, measuring approximately 35 km long and 2 km wide (Vasconcelos, 2000). It is filled by coal-rich rocks belonging to the Karoo Supergroup, which unconformably overlie older Proterozoic formations (Vasconcelos, 2000). This coal-bearing Karoo Supergroup forms part of the east–west trending Zambezi Basin. The Zambezi Basin itself is segmented into several disconnected sub-basins—including Chicôa-Mecúcoè, Sanângoè-Mefídézi, Moatize, Muarazi, and Minjova, with extensions to Ncondezi and Mutarara (Vasconcelos, 2012). This fragmentation is a direct result of Jurassic and Cretaceous extensional tectonics and subsequent erosion (Hancox, 2016). The Karoo Supergroup in this region comprises four primary stratigraphic units, listed from oldest to youngest: the Vúzi, Moatize, Matinde, and Cádzi Formations (Vasconcelos, 2012). Notably, Fernandes et al. (2015) did not identify the Cádzi Formation in their study, suggesting it may have been removed through erosion. The Moatize Formation (Fig. 1) consists of interbedded carbonaceous mudstones, pure mudstones, rhythmites, sandstones, and cyclic coal deposits (Bicca et al., 2018). These are interpreted as fluvial and lacustrine sediments deposited in a wet, temperate climate (Mugabe, 1999). This formation contains bituminous coal with a low to medium volatile matter content (1.3–1.7% Rr) (Fernandes et al., 2015).

Coal mining in Moatize started in the early twentieth century with small, open-pit operations. By 1940, underground mining was producing around 10,000 tons yearly, with output peaking in 1975 at 575,000 tons (MAE, 2014). Large-scale production, particularly open-pit mining, started in 2011. Subsequently, four mines became operational, leading to a production volume of 15.2 million tons (Mt) in 2018, with the majority of this coal designated for export (Egger et al., 2025). Currently, the Moatize district operates three active mines: the Moatize Mine, which Vulcan (a subsidiary of Vale Moçambique) has run since 2011; the Benga Mine, managed by the ICVL (International Coal Ventures Private Limited**)** consortium; and the Minas Moatize Mine, explored by Beacon Hill Resources. Both the Benga Mine and Minas Moatize Mine have been operational since 2012.

The Moatize coalfield contains several promising coal layers suitable for both coking and thermal purposes, which are stratigraphically arranged and interbedded with pelitic rocks, such as shale, siltstone, and minor sandstone units (Vasconcelos, 2000). These valuable resources are organized into six productive complexes: Bananeiras, Chipanga, Sousa Pinto, Intermédia, Grande Falésia, and André, whose specific characteristics are detailed in Table 1 (Lakshminarayana, 2015). The geological sequence gently slopes toward the basin’s center, with dip angles ranging from 8 to 15 degrees (Vasconcelos et al., 2014). Based on their dry, mineral-matter-free volatile matter percentage, the Moatize coals were classified by the American Society for Testing and Materials (ASTM) as ranging from medium volatile bituminous to semianthracite (Lakshminarayana, 2015; Sampaio et al., 2020).

**Table 1:** Seams raw coal quality (air dried basis) of Moatize (Lakshminarayana, 2015; Sampaio et al., 2020)

Properties	Coal seam					
	Souza Pinto	Chipanga	Bananeiras	Intermédia	Grande Falésia	André
% Moisture	0.84	0.85	0.92	1.04	1.04	1.2
% Ash	56.07	37.94	38.09	41.76	43.74	36.18
% Volatile matter	12.15	16.27	16.74	16.3	16.75	18.68
% Fixed carbon	30.94	44.94	44.24	40.89	38.46	43.94
% Total	100	100.00	100	100	100	100
% Sulphur	1.1	4.12	4.03	3.77	3.8	3.81
MJ/kg	13.8	17.62	17.57	16.45	15.62	21.37
Btu/lb	5934	8930	8918	8381	8067	9189

Mining operations in Moatize regularly release untreated, polluted water PTEs into the Zambezi River basin (Pondja et al., 2017b). This practice poses a serious threat to water quality and public health. A key source of this contamination is the unprocessed coal (Run-of-Mine, or ROM) stockpiles, which contain raw coal and various impurities. When exposed to the elements, these stockpiles become active sources of pollution. The presence of pyrite in Moatize’s coal (Vasconcelos, 1995) makes AMD highly probable, which in turn mobilizes PTEs into both groundwater and surface water. In addition, runoff from stockpiles, along with unprocessed coal dumped at mine sites and coal dust from blasting (Rouhani et al., 2023), introduces these PTEs into local waterways. The impacts of this pollution are often visible as black particles in rivers and community water taps.

Local community practices also worsen the issue. The ash from exposed coal used in brick kilns is left on the ground, where it can be leached by water or carried by wind and animals into rivers. This is especially concerning, as recent studies have shown that some fly ash contains high levels of water-soluble Chromium VI (Cr (VI)), a potent carcinogen (Finkelman, 2024). The combined effect of mining operations, ROM materials, and community practices leads to widespread and long-lasting water contamination, which may be a factor exacerbating disease outbreaks in nearby communities. This environmental threat occurs against a background of high regional mortality from malaria, tuberculosis, pneumonia, diarrhea, circulatory conditions, and cancers (de Tete, 2015). Specifically, among adolescents in mining areas, Cambaco et al. (2025) identified sexually transmitted, respiratory tract, and diarrheal infections, alongside malaria, as the key health concerns, and that the community perceives that mining exacerbates negative health effects.

## Materials and methods

### Sampling of coal, ash, surface, and groundwater

Three coal samples were collected from three outcropping layers accessible to local communities, and eight samples were collected from run-of-mine (ROM) material provided by mining companies. Approximately 5 kg of coal was collected from each outcropping layer from locations convenient for sampling. Five ash samples from laboratory-combusted coal (two from the mining site, three from outcropping layers) were also assessed in this study.

A total of 30 water samples were collected over 2 years (2023–2024), comprising 24 surface water samples and 6 groundwater samples, with sampling points located both upstream and downstream of the mine environment. The sampling strategy aimed to characterize and compare water quality during the hydrological extremes represented by the dry and wet seasons, which capture extremes of concentration and dilution. Twenty samples (14 surface water and all 6 groundwater samples) were collected during the 2023 dry season (Fig. 1), while the remaining 10 samples were surface water collected during the 2024 rainy season. The samples were gathered along the Zambezi, Revúboè, Moatize, Murongozi, and Muarazi Rivers. Sampling locations were strategically chosen based on their proximity to inhabited areas and coal mines. Groundwater was obtained from shallow hand-dug wells (15–150 cm deep) only where the surface riverbed was dry.

The physicochemical parameters (pH, temperature [T], total dissolved solids [TDS], and electrical conductivity [EC]) of the unfiltered water were measured in situ using a HANNA HI 9828 portable multiparameter probe. Samples were collected using a plastic bucket, rinsed with site water to prevent contamination, stored in labeled polyethylene plastic bottles, and preserved via refrigeration at 4 °C ± 2 °C before laboratory analysis.

The resultant asymmetry in sampling—specifically the larger sample in the 2023 dry season and the exclusion of groundwater in the 2024 rainy season—was necessitated by logistical and resource-based constraints. An extensive 2023 dry-season collection was maximized due to available preliminary funding and favorable field accessibility. In contrast, 2024 rainy-season fieldwork was severely restricted by floods and associated safety concerns. Although asymmetric, this regime successfully generated a robust dataset for characterizing the two primary target hydrological seasons.

### Laboratory analysis and quality control

All coal, coal ash, and water samples were analyzed for elemental composition (Ba, Co, Cr, Cu, Mn, Mo, Pb, Sr, V, U, and Zn) using inductively coupled plasma mass spectrometry (ICP-MS). Cations, including Ca^2+^, Mg^2+^, K⁺, Na⁺ were analysed using ICP-MS following the procedures described by Balaram et al. (2023). Anions, including NO_3_⁻, Cl⁻, SO_4_^2⁻^, HCO_3_⁻, and F^¯^ were analysed using ion chromatography (IC) following the procedures described by Michalski (2024). Scanning Electron Microscopy coupled with Energy Dispersive Spectrometry (SEM–EDS) was used to identify the elements in the coal samples. The analysis was carried out at the University of Johannesburg.

To ensure data reliability, stringent quality assurance and quality control (QA/QC) protocols were implemented throughout all stages, from sample collection to laboratory analysis. Contamination was stringently controlled by subjecting all materials to acid-washing, thorough rinsing with Milli-Q water, and air-drying, while procedural blanks were employed to account for potential background contamination. Analytical precision was evaluated using triplicate samples and replicates, whereas accuracy was determined by analyzing certified reference materials, standard solutions, and reagent blanks. Laboratory accuracy and precision were further confirmed with duplicate sample analysis and regular standard solution checks. All equipment underwent calibration and inspection. Each analysis was performed in triplicate, and the mean value was reported. The process was repeated until an accuracy of 95% and a precision of 5% were achieved. The results were validated using SRM 3120B following the procedure described by Bridgewater (2017). The data’s reliability was supported by several key metrics, which are detailed in Table 2. The percentage recoveries for all tested PTEs, including Ba, Co, Cr, Cu, Mn, Mo, Ni, Pb, Sr, V, NO_3_⁻, and F⁻, were within the acceptable range of 80–120%. Furthermore, a strong correlation between instrument response and analyte concentration was demonstrated by R^2^ values that all exceeded 0.99. The limit of detection (LOD) and limit of quantification (LOQ) for each PTE are also listed in Table 2.

**Table 2:** Quality assurance and control results for analyzed PTEs

	%R	R^2^	LOD (mg/L)	LOQ (mg/L)
NO_3_^−^	91.5	0.9991	6.00E–03	1.82E–02
F^−^	99.03	0.9999	1.00E–03	3.03E–03
Cl^−^	94.47	0.9994	1.40E–02	4.24E–02
HCO_3_^−^	103.01	0.9995	1.20E–02	3.64E–02
Ca	97.18	0.9997	7.00E–01	2.12E + 00
Na	97.57	0.9998	5.00E–01	1.52E + 00
K	105.01	0.9999	3.00E–02	9.09E–02
Mg	99.25	0.9998	2.00E–03	6.06E-03
Ba	100.11	0.9998	1.00E–03	3.03E–03
Co	101.66	0.9999	1.00E–03	3.03E–03
Cr	94.34	0.9995	2.00E–03	6.06E–03
Cu	97.60	0.9999	2.00E–03	6.06E–03
Mn	96.52	0.9999	1.00E–03	3.03E–03
Mo	92.16	0.9998	1.00E–03	3.03E–03
Ni	99.48	0.9999	1.00E–03	3.03E–03
Pb	99.47	0.9999	1.00E–02	3.03E–02
Sr	100.43	0.9999	4.00E–03	1.21E–02
V	97.83	0.9998	1.00E–03	3.03E–03
Zn	85.35	0.9999	1.00E–03	3.03E–03

%R, Percent Recovery; R^2^, Coefficient of determination; LOD, Limit of detection; LOQ, Limit of quantification

## Data analysis

### Pollution assessment indices for water quality

The following two water pollution indices: i) water quality index (WQI) and ii) pollution index (PI) were calculated to determine the pollution status of surface and groundwater, to assess the pollution levels of different drinking water sources in the study area.

The WQI is a model utilized to evaluate the suitability of surface and groundwater quality for domestic use (Manna & Biswas, 2023). The WQI was calculated using parameters such as pH, Electrical conductivity (EC), Turbidity, Total dissolved solids (TDS), and Ca concentration, as illustrated in Table 3. The proportionality constant K was calculated using Eq. 1.

1 $$K = \frac{1}{{\sum {\raise0.7ex\hbox{$1$} \!\mathord{\left/ {\vphantom {1 {Sn}}}\right.\kern-0pt} \!\lower0.7ex\hbox{${Sn}$}}}}$$

where *S*_*n*_ is the recommended standard quality value of a *n*^th^ parameter for the drinking water. The unit weight (W_n_) for each water quality parameter was calculated using Eq. 2.

**Table 3:** Parameters used for WQI calculation

Water quality parameter	Unit	Sn	K	Wn	V_ideal_
pH	–	8.5	2.973414	0.349813	7
EC	μs/cm	300	2.973414	0.009911	0
Turbidity	NTU	5	2.973414	0.594683	0
TDS	mg/L	500	2.973414	0.005947	0
Ca	mg/L	75	2.973414	0.039646	0

Sn is the standard permissible value for drinking water (WHO); K is the proportionality constant; Wn is the relative weight; V_ideal_ is the ideal value

2 $$Wn= \frac{K}{Sn}$$

The quality rate scale (Qi) was determined using Eq. 3.

3 $$Qi= \frac{Vn-Videl}{Sn-Videal} \times 100$$

where Vn represents the measured concentration of the n^th^ variable, V_ideal_ is the ideal concentration of the individual variable in freshwater (V_ideal_ = 0) V_ideal_ of pH is taken as 7.0. The WQI was calculated by using Eq. 4.

4 $$WQI= \frac{\sum_{i=1}^{n}(Qi \times Wn)}{\sum_{i=1}^{n}Wn}$$

These indices were used to classify the groundwater into excellent water quality (WQI: 0–25), good water quality (WQI: 26–50), poor water quality (WQI: 51–75), very poor water quality (WQI: 76–100), and unsuitable for drinking (WQI: > 100) (Siganga et al., 2023).

*The pollution index (PI)* calculates the overall contamination of PTEs in water resources (Sarhat & Al‑Obaidi, 2023). The PI index values can be classified into three categories as shown below. The PI was computed using Eq. 5.

5 $$\mathrm{PI}= \frac{{\sum }_{\mathrm{i}=1}^{\mathrm{n}}\mathrm{WiQi}}{{\sum }_{\mathrm{i}=1}^{\mathrm{n}}\mathrm{Wi}}$$

where Wi is the unit weightage of the i^th^ parameter, Qi is the sub-index of the i^th^ parameter, and n is the total number of parameters measured. The unit Weight (Wi) and the recommended standard (Sn) are both inversely proportional to each other. PI is classified as < 15 low, 15–30 Medium, and > 30 high (Sarhat & Al‑Obaidi, 2023). The sub-index (Qi) of each element is calculated by using Eq. 6.

6 $${\mathrm{Qi}} = \mathop \sum \limits_{{{\mathrm{i}} = 1}}^{{\mathrm{n}}} \frac{{\left| {{\mathrm{Mi}} - {\mathrm{Ii}}} \right|}}{{{\mathrm{Sn}} - {\mathrm{Ii}}}} \times 100$$

where Mi is the concentration of individual PTEs, Ii is the ideal value of the (i^th^) PTE in (ppb), and Sn is the standard permissible limit of the (i^th^) PTEs. The ideal values for all the PTEs adopted are zero.

### Water geochemistry: evolution and controlling processes

This study utilized Piper and Gibbs diagrams to identify the primary hydrochemical water type and the natural factors influencing groundwater composition. Hydrogeochemical facies characterise the water’s chemical nature within the hydrological systems, which is aided by Durov’s plot (1948). The Piper and Durov plots were generated using Grapher® software. Statistical analysis was performed using SPSS and Excel.

### Geospatial and statistical analysis

The geographic coordinates of the sampling locations were recorded using a Garmin GPSMAP 64 s unit and subsequently plotted on the geological map of the study area. The data were statistically analyzed using Microsoft Excel, and Pearson correlation coefficients (r) were calculated to determine the relationships between PTEs and physico-chemical parameters.

### Human health risk assessment

Risk assessment quantifies the probability and magnitude of adverse health impacts caused by exposure to environmental hazards. For potentially toxic elements in water, human health risks primarily arise from oral ingestion, inhalation, and dermal contact (Salami et al., 2025). Non-carcinogenic health risks for both adults and children from waterborne elements were assessed using the UEPA (2004) methodology. This involved calculating the Hazard Quotient for oral intake (HQ_oral_) (Eq. 7) and Hazard Quotient for dermal exposure** (**HQ_dermal_) (Eq. 8). Both were based on wet and dry season water

7 $${\mathrm{HQ}}_{{{\mathrm{oral}}}} = {\text{ CDI}}/{\mathrm{RfD}}$$

8 $${\mathrm{HQ}}_{{{\mathrm{dermal}}}} = {\text{ DAD}}/{\mathrm{RfD}}$$

where CDI denotes the oral chronic daily intake, while DAD represents the dermal absorption dose. The oral reference dose (RfDo) is the health benchmark for daily chemical exposure that is unlikely to cause adverse health effects over a lifetime for a given human population (FAO/WHO, 2013). The specific RfDo values used in this study were: NO_3_⁻ (1.6 mg/kg day) (Morovati et al., 2024), Mn (0.24 mg/kg day), Mo (0.005 mg/kg day) (Xiao et al., 2019), V (0.005 mg/kg day) (EPA, 2011), Se (0.00004 mg/kg day), Pb (0.0035 mg/kg day) (FAO/WHO, 2013), and Cu (0.004 mg/kg day) (Taylor et al., 2020). The Dermal Reference Dose (RfDd) is defined as an estimate of the daily quantity of a chemical that a person could be exposed to via dermal absorption for a lifetime without significant risk (EPA, 2011). The RfDd values mg/kg-day) applied were Mn (0.00096 mg/kg day) (Xiao et al., 2019), Cu (0.001 mg/kg day) (Zheng et al., 2015), and Mo (1.9 mg/kg day). Pb (0.0035 mg/kg day) (FAO/WHO, 2013), and V (0.005 mg/kg day) (EPA, 2011).

Established mathematical models were used to quantify exposure via both oral intake (ingestion) and skin absorption (dermal contact). Equations (9) and (10) were used to determine CDI and DAD, both in mg/kg/day, respectively.

9 $$\mathrm{CDI}=\mathrm{C}\times \mathrm{EF}\times \mathrm{ED}\times \mathrm{IR}/\mathrm{ABW}\times \mathrm{AET}$$

10 $$\begin{aligned}\mathrm{DAD}&=\mathrm{C}\times \mathrm{TC}\times \mathrm{Ki}\times \mathrm{CF}\times \mathrm{EV}\times \mathrm{ED}\\ & \quad \times \mathrm{EF}\times \mathrm{SSA}/\mathrm{ABW}\times \mathrm{AET}\end{aligned}$$

where C (mg/L) is the concentration of potential toxic elements in water. The exposure frequency (EF) is set at 365 days/year for both adults and children. The exposure duration (ED) is assumed to be 70 years for adults and 15 years for children. The ingestion rate (IR) is defined as 2.5 L/day for adults and 1.0 L/day for children. The dermal contact duration (TC) is 0.4 h/day (USEPA, 1997). The dermal adsorption coefficient (Ki) is 0.001 cm/h (Ayejoto et al., 2024). The conversion factor (CF) is 0.00134 (USEPA,^1997^). The bathing frequency (EV) is once a day (times/ day) (USEPA, 1997). The skin surface area (SSA) is 16,600 cm^2^ for adults and 12,000 cm^2^ for children (USEPA, 1997). In addition, the average exposure time (AET) is specified as 25,550 days for adults and 5,475 days for children (FAO/WHO, 2013). The average body weight (ABW) used is 70 kg for adults and 15 kg for children (USEPA, 1997).

To determine the overall potential for non-carcinogenic health effects arising from exposure to a combination of heavy metals in water, the hazard index (HI) was calculated for a suite of heavy metals, following the health risk assessment guidelines established by the EPA (2011) and using the subsequent method Eq. 11.

11 $$\mathrm{HI}={\sum }_{\mathrm{i}=1}^{\mathrm{n}}\mathrm{HQi}$$

where HQi represents the hazard quotient for a single metal, while HI denotes the cumulative hazard index calculated for all 15 metals investigated in this study.

A hazard quotient (HQ) below unity suggests negligible potential non-carcinogenic risks to the population, whereas an HQ of one or more indicates a considerable potential for such risks (USEPA, 2004). A hazard index (HI) value greater than one (HI > 1) denotes a possible adverse impact on human health, with higher values signifying a greater degree of hazard (USEPA, 2004).

## Results

### Assessment of coal, ash, and water properties

#### Geochemical composition of coal and coal ash

This study compared the average elemental concentrations of coal and ash samples from Moatize to global benchmarks for hard coals and ash, using data from Ketris & Yudovich (2009), Marove et al. (2020), and Vasconcelos et al. (2014).

In coal samples, the concentrations of Ba, Cr, Cu, Mn, Pb, V, and Zn were higher than the benchmarks from Ketris & Yudovich (2009). The concentrations of Mn, V, and Zn also exceeded those reported by Marove et al. (2020). For ash samples, all elements analyzed had concentrations greater than those reported by Vasconcelos et al. (2014), and Co and Cr concentrations surpassed the global benchmarks from Ketris & Yudovich (2009).

A detailed comparison is presented in Table 4, where the counts of samples exceeding the Ketris & Yudovich (2009) benchmarks are highlighted in bold. The full chemical composition of all analyzed coal and ash samples from Moatize is presented in Table 5.

**Table 4:** Mean concentration of coal and ash from Moatize compared to Ketris and Yudovik (2009), in mg/kg

	Coal					Ash				
	Mean	N SA	Marove et al. (2020)	N SA	Ketris & Yudovik (2009)	Mean	N SA	Vasconcelos et al. (2014)	N SA	Ketris & Yudovik (2009)
As	3.5	0	2.2	0	9.0 ± 0.7	–	0	nd	0	46 ± 5
Ba	**253.8**	11	nd	–	150 ± 10	630.3	0	476	0	980 ± 60
Co	5.6	5	**7**	1	6.0 ± 0.2	**47.4**	5	9.9	0	37 ± 2
Cr	**40.5**	11	**45.5**	5	17 ± 1	**167.0**	5	23.2	0	120 ± 5
Cu	**18.2**	4	**19.8**	4	16 ± 1	58.2	0	32.7	0	nd
Hg	0.07	1	nd	–	0.10 ± 0.01	0.1	–	nd	0	0.1
Mn	**112.9**	4	**82.2**	2	71 ± 5	426.8	0	37.4	0	430 ± 30
Mo	3.8	11	nd	nd	2.1 ± 0.1	10.1	0	nd	0	14 ± 1
Ni	13.5	0	**24.1**	5	17 ± 1	41.8	0	20.8	0	100 ± 5
Pb	**12.3**	8	**14.7**	5	9.0 ± 0.7	47.0	0	nd	0	55 ± 6
Se	0.5	nd	0.6	0	nd	nd	–	nd	0	nd
Sr	**105.5**	6	nd	nd	100 ± 7	**871.3**	2	151.9	0	730 ± 50
U	**2.3**	8	nd	nd	1.9 ± 0.1	6. 9	0	nd	0	15 ± 1
V	**58.8**	11	**39.7**	6	28 ± 2	**179.0**	3	46.2	0	170 ± 10
Zn	**30.6**	6	26.4	2	28 ± 1	128.8	0	47	0	170 ± 10

**Table 5:** Chemical composition of coal and ash samples from Moatize, in mg/kg

Elements	Coal											Ash				
	C1	C2	C3	C4	C5	C6	C7	C8	C9	C10	C11	A1	A2	A3	A4	A5
As	3.97	4	2.21	6.1	1.4	0.98	2.39	3.29	1.59	8.36	4.16	–	–	–	–	–
Ba	**233.4**	**230.4**	**253.9**	**420.2**	**233.5**	**151.3**	**209.0**	**190.0**	**255.9**	**240.4**	**267.2**	516.2	826.1	549.0	632.6	627.8
Co	5.7	**6.4**	**6.2**	**7.4**	3.8	4.3	**6.8**	4.3	3.8	5.2	5.9	**45.6**	**56.7**	**40.4**	**42.3**	**50.8**
Cr	**31.6**	**31.21**	**34.47**	**30.63**	**28.84**	**24.47**	**39.6**	**45.6**	**33.5**	**83.1**	**62.6**	**174**	**186**	**146**	**164**	**165**
Cu	15. 7	15.3	**19.9**	**20.7**	15.6	**17.6**	13.9	12.4	11.1	**34.0**	**23.8**	59.3	50.4	48.2	66.9	62.9
Hg	0.06	0.07	0.05	0.11	0.03	0.06	–	–	–	–	–	–	–	–	–	–
Mn	56.6	58.4	**117.4**	**97.2**	42.0	13.5	**285.1**	**430.0**	25.6	45.1	70.7	1229.0	17.5	111.7	88.9	186.5
Mo	**2.9**	**3.2**	**3.3**	**3.3**	**3.1**	**3.2**	**2.6**	**3.2**	**2.7**	**3.6**	**3.3**	9.3	8.4	12	11.3	8.2
Ni	15.5	16.6	14.9	16.7	11.4	11.6	10.3	14.2	10.3	14.5	12.8	44.8	56.9	44.9	28.6	33.8
Pb	**10.5**	**10.6**	**10.9**	**15.3**	9.4	9.4	**18.8**	7.1	**12.6**	**15.8**	**15.2**	80	28.9	55	31.2	40.1
Se	0.44	0.54	0.51	0.97	0.32	0.54	0.27	0.24	0.27	0.54	0.37	–	–	–	–	–
Sr	79.0	85.1	93.1	**132.4**	**129.2**	**113.9**	98.2	83.1	**109.0**	**103.2**	**108.2**	**958.2**	678.3	**1735.0**	515.4	469.8
U	**2.1**	**2.3**	**2.33**	**2.63**	**2.03**	**2.43**	1.77	1.52	1.56	**3.1**	**2.9**	7.5	6.2	6.8	6.0	7. 9
V	**59.9**	**66.4**	**66.8**	**60.8**	**55.5**	**57. 7**	**47.7**	**51.2**	**33.0**	**80.5**	**67.2**	**206.1**	**209.1**	144.0	159.0	**177.2**
Zn	18.3	18.9	**33.8**	**44.4**	17.5	24.4	**49.2**	**49.5**	21.5	**29.6**	**29.9**	211.1	202.0	93.8	58.2	79.0

bold text is used to identify sample counts that exceed the benchmarks established by Ketris and Yudovich (2009)

#### SEM/EDS of coal

**Fig. 2:**
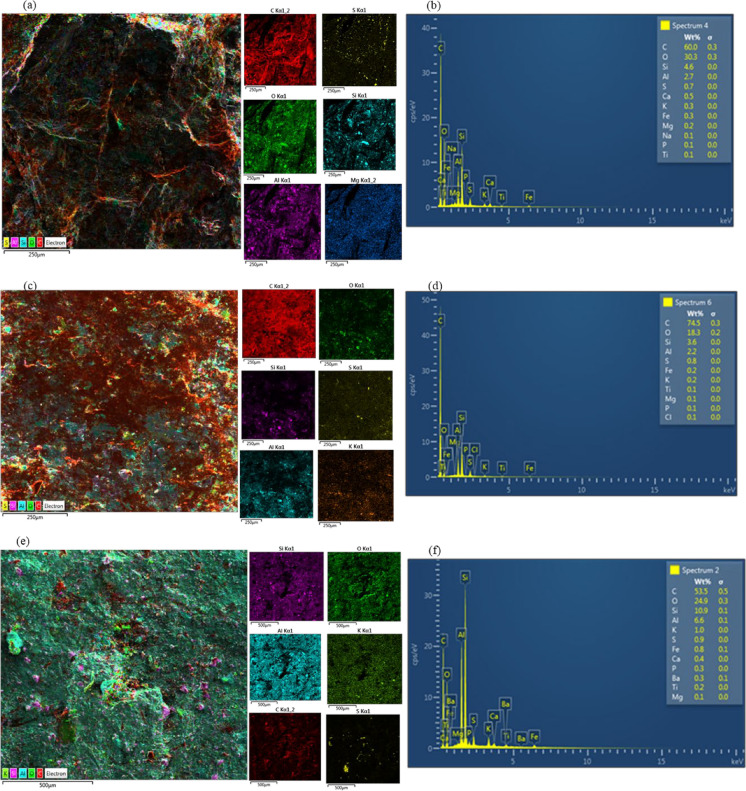
SEM/EDS images of elemental composition of Lower Chipanga (2**a**-**b**), Upper Chipanga (2**c**-**d**), and Bananeiras (2**e**-**f**) seams from the Moatize coal field

Scanning Electron Microscopy with Energy Dispersive Spectrometry (SEM/EDS) was employed to analyze the elemental composition of the Lower Chipanga (LC), Upper Chipanga (UC), and Bananeiras (Bn) coal seam samples (Fig. 2). Across all samples, the most abundant elements, in decreasing order, were carbon (C), oxygen (O), silicon (Si), and aluminum (Al). Distinct elemental variations were observed. The LC seam featured a set of elements including sulfur (S), calcium (Ca), potassium (K), iron (Fe), magnesium (Mg), sodium (Na), phosphorus (P), and titanium (Ti). In contrast, the UC seam lacked Ca and Na, but was the sole seam to contain chlorine (Cl). The Bn seam mirrored the LC seam’s composition, except Na, and was uniquely characterized by the presence of barium (Ba).

### Physical parameters and geochemical composition of water

- Physicochemical parameters

The physical characteristics of water from Moatize were compared to the drinking water limits set by the WHO (2021) and Mozambique’s Water Quality Legislation (MWQL), which is outlined in Ministerial Diploma n.° 180/2004 (Table 6). Physical water parameters in Moatize demonstrated seasonal variations, with both wet and dry season data revealing adherence to some drinking water standards while exceeding others.

**Table 6:** Average physicochemical characteristics of Moatize’s water

	Surface water								Ground water				
Parameter	Wet				Dry								
	Min	Max	Mean	SNA	Min	Max	Mean	SNA	Min	Max	Mean	SNA	WHO (2021); MWQL
*Physicochemical parameters and chemical composition of surface and groundwater*													
EC (μS/cm)	34.5	1269.0	674.3	8	158.8	1565.0	655.7	7	926.0	3680	2702	6	400; 2000
pH	7.6	8.4	8.1	0	7.5	8.3	7.9	0	7.2	8.2	7.7	0	6.5–8.5
T(^o^C)	24.6	25.0	25.0	0	25.0	25.4	25.1	0	25.0	25.3	25.1	0	30
TDS (mg/L)	102.0	1836.0	576.8	2	79.0	783.0	332.4	0	154.0	467.0	268.3	0	1000
Turbidity	5.9	97.0	26.0	14	0.2	348.0	64.9	2	7.1	18.0	11.9	6	5
Hardness	31.0	236.0	112.9	0	31.0	236.0	112.9	0	29.0	38.0	33.0	0	–
Na (mg/L)	8.5	176.0	74.7	0	5.5	180.0	53.9	0	73.0	450.0	307.5	3	200
K (mg/L)	2.3	11.3	4.1	1	2.4	10.2	5.1	1	4.2	11.1	6.9	1	10
Ca (mg/L)	14.5	130.0	52.9	4	12.6	94.8	45.1	4	116.0	218.0	152.0	6	50
Mg (mg/L)	4.8	57.5	33.0	4	4.5	55.8	28.2	2	37.3	135.0	94.7	4	50
Cl (mg/L)	33.4	191.0	81.0	0	0.6	144.2	39.5	0	19.4	554.0	240.5	3	250
HCO_3_ (mg/L)	678.0	813.0	740.6	14	567.0	727.0	655.4	10	743.0	843.0	785.7	6	350
SO_4_ (mg/L)	65.4	97.6	79.0	0	58.2	79.7	68.0	0	778.0	1722.0	1321.8	6	200
NO_3_ (mg/L)	1.3	9.3	4.8	0	0.1	3.2	1.1	0	8.6	1763	562.7	2	50
*Chemical composition of potentially toxic elements (PTEs)*													
	Surface					Groundwater							
	Min	Max	Mean	SNA		Min	Max	Mean	SNA				
Cu (mg/L)	0.003	1.2	0.418	1		0.000	0.021	0.008	0				2.0; 1.0
Se (mg/L)	0.001	0.036	0.011	11		0.017	0.024	0.020	6				0.04; 0.01
Si (mg/L)	3.64	123	31.39	2		26.43	123.0	55.180	6				29.9
Mo (mg/L)	0.001	0.034	0.009	0		0.001	0.237	0.119	1				0.07
Mn (mg/L)	0.001	0.237	0.067	2		0.001	0.030	0.016	0				0.4; 0.1
Pb (mg/L)	0.001	0.015	0.006	3		0.000	0.020	0.010	2				0.01
V (mg/L)	0.001	0.041	0.018	6		0.018	0.042	0.031	5				0.021

During the dry season, water temperatures ranged from 24.5 to 25.1 °C in surface water and from 24.7 to 24.9 °C in groundwater, which is within the 30 °C limit recommended by the WHO. pH values in both surface (7.6–8.4) and groundwater (7.2–8.2) fell within the WHO and MWQL permissible range of 6.2–8.5. However, Turbidity was a universal concern, as all dry-season samples exceeded the maximum allowable limit of 5 NTU. The Zambezi River had the highest overall turbidity and Total Dissolved Solids (TDS) readings. EC showed significant variation, ranging from 34.5 to 1269 μS/cm in surface water and 926–3680 μS/cm in groundwater. Numerous samples surpassed the WHO threshold of 400: four surface water samples from the Moatize River, two from the Murongozi River, two from the Murazi River, and all groundwater samples. Additionally, two groundwater samples exceeded the stricter MWQL limit of 2000 μS/cm. All sampled rivers exhibited longitudinal degradation along the stream, where Turbidity, EC, and TDS concentrations were lower upstream but increased downstream. The Zambezi exhibited the greatest variance in Turbidity and TDS, and the Moatize River recorded the highest overall EC variation, with all three parameters markedly increasing downstream.

During the wet season, water temperatures ranged from 24.7 to 25.4 °C, which is within the 30 °C limit recommended by the WHO. pH values were consistent with dry season results, ranging from 7.5 to 8.2, well within the permissible 6.2–8.5 limits. Turbidity showed a broad range from 0.24–348 NTU, peaking in the Moatize River. Mean EC values ranged from 155 to 158.8 μS/cm, with all river waters exceeding the WHO threshold of 400 μS/cm. TDS levels varied from 79 to 783 mg/L, but all remained below the MWQL maximum limit of 1000 mg/L for drinking water. The pattern of longitudinal degradation persisted: Turbidity, EC, and TDS concentrations increased downstream along all rivers. The Zambezi River showed the highest overall turbidity but had the lowest EC and TDS values. In contrast, the Moatize River recorded the highest overall EC and TDS concentrations and clearly showed that all three parameters have increased downstream. The Muarazi River has shown this longitudinal increase, and the Murongozi River had the lowest overall turbidity levels. Such increases along the river course are expected and reflect runoff from natural and anthropogenic sources.

b) Geochemical composition

Average element concentrations in Moatize’s water samples are presented in Table 6. These levels were assessed against guidelines from the WHO (2021), MWQL, the Agricultural & Environmental Services Laboratories (AEAL) for Silicon, and the Environmental Working Group (EWG) for Vanadium.

The Concentration of Mo in surface water ranged from 0.030 to 0.34 mg/L during the dry season and 0.01–0.016 mg/L in the wet season. The concentration of Mo in groundwater varied from 0.03 to 0.034 mg/L, all below both the WHO and MWQL threshold of 0.07 mg/L.

Concentrations of Pb were above the WHO and MWQL limit of 0.01 mg/L in dry season water samples from Moatize and Muarazi Rivers, one sample from the Zambeze River, and in two groundwater samples from the Moatize River. However, the concentration of Pb varied from 0.001 to 0.015 mg/L during the dry season, and from 0.003 to 0.011 mg/L in the wet season mg/L. Concentrations in groundwater ranged from 0.001 to 0.016 mg/L. The Se concentrations in surface water ranged from 0.005 to 0.036 mg/L during the dry season and from 0.010 to 0.013 mg/L during the wet season. Groundwater concentrations ranged from 0.017 to 0.024 mg/L. However, surface water from the Moatize, Revúboè, Murongozi, and Zambeze Rivers in the dry season and from the Moatize and Murongozi Rivers in the wet season. All the groundwater revealed a concentration of Se above the MWQL threshold of 0.01 mg/L but remained below the WHO limit of 0.04 mg/L. Concentration of Si in surface water ranged from 3.64 to 123 mg/L in the dry season, 7.80–24.87 mg/L in the wet season, and from 26.43 to 123.0 mg/L in groundwater. Only surface water from the Murongozi River and groundwater from all sampled rivers had Si concentrations exceeding the AEAL limit of 29.9 mg/L. Mn concentration of surface water ranged from 0.001 to 0.237 mg/L in the dry season, from 0.001 to 0.005 mg/L in the wet season, and from 0.01 to 0.030 mg/L in groundwater, with surface water from the Muarazi River surpassing 0.1 mg/L settled by MWQL. V concentration in surface water ranged from 0.001 to 0.041 mg/L during the dry season and from 0.001 to 0.020 mg/L during the wet season. The V concentration in groundwater ranged from 0.018 to 0.048 mg/L. All the rivers had water samples from the dry season and groundwater that showed concentrations of V above 0.021 mg/L, settled by EWG. Cu concentration ranged from 0.02 to 0.028 mg/L in the dry season, from 0.003 to 0.911 mg/L in the wet season, from surface water, and from 0 to 0.021 mg/L in groundwater. All of the surface water and groundwater analyzed had Cu concentrations below the permissible limits for drinking water set by the WHO (2.0 mg/L) and the MWQL (1.0 mg/L).

Anions: Chloride (Cl⁻) concentrations in surface water ranged from 33.4 to 130.0 mg/L in the dry season, sharply increasing to 0.62–144.19 mg/L in the wet season. Groundwater consistently showed the highest levels, ranging from 19.4 to 554.0 mg/L, with only groundwater samples exceeding the WHO limit of 250 mg/L. Bicarbonate (HCO_3_^−^) levels in water consistently surpassed the WHO’s 350 mg/L guideline in all samples. Dry season concentrations in surface water were 678–813 mg/L, while wet season levels were slightly lower at 567–727 mg/L. Groundwater samples exhibited HCO_3_⁻ concentrations between 743 and 843 mg/L. Sulfate (SO_4_^2−^) surface water concentrations were 65.4–97.6 mg/L in the dry season and 58.2–79.7 mg/L in the wet season. However, all groundwater samples had significantly higher concentrations (778–1763 mg/L), exceeding the WHO limit of 200 mg/L. Nitrate (NO_3_^−^) concentrations varied from 1.31 to 9.25 mg/L in the dry season, 0.13–3.24 mg/L in the wet season, and 8.61–1763 mg/L in groundwater. Two groundwater samples exceeded the WHO limit of 50 mg/L.

Cations: Na concentrations in surface water ranged from 8.49 to 176 mg/L in the dry season and 5.50–180.0 mg/L in the wet season. Groundwater showed a significant increase, with concentrations ranging from 73.03 to 450.0 mg/L. Notably, two surface water samples and four groundwater samples exceeded the WHO and MWQL limit of 200 mg/L. K concentrations were generally lower, ranging from 2.34 to 11.28 mg/L in dry season surface water, 2.43–10.18 mg/L in wet season surface water, and 4.15–11.09 mg/L in groundwater. One sample from each source (surface water and groundwater) surpassed the WHO limit of 10 mg/L. Mg concentrations in dry season surface water ranged from 4.76 to 57.54 mg/L, dropping sharply in the wet season to 4.54–55.76 mg/L. Groundwater exhibited increased Mg levels, ranging from 37.7 to 135.0 mg/L. Six surface water samples (across both seasons) and four groundwater samples exceeded the WHO and MWQL limit of 50 mg/L. Ca concentrations in surface water varied from 15.0 to 130.0 mg/L in the dry season and from 12.59 to 94.75 mg/L in the wet season. Groundwater concentrations were notably higher, ranging from 116.0 to 218.0 mg/L. All groundwater samples and eight surface water samples exceeded the 50 mg/L limit set by both the WHO and MWQL.

The longitudinal distribution for ions and metals showed significant seasonal reversal between the dry and wet seasons. During the dry season, Na, Mg, and Si were generally high upstream across most rivers. SO_4_ was consistently high upstream in all rivers. Pb was low upstream in Moatize, Muarazi, and Zambezi. NO_3_ was high downstream in Moatize, Zambezi, and Revúboè; conversely, the Muarazi and Murongozi rivers had high concentrations of NO_3_ upstream. K and Cl showed mixed high upstream or downstream patterns depending on the river. During the wet season, the previous trend was largely reversed, to higher concentrations of Na, Ca, Mg, Cl, and SO_4_ downstream in the Moatize and Muarazi rivers. Si showed a notable shift, being high upstream in the Moatize but high downstream in the Muarazi. K also split, being high downstream in the Moatize but high upstream in the Muarazi. NO_3_ was high downstream in the Moatize and high upstream in the Muarazi. Pb and Se concentrations were generally low upstream.

### Hydrogeochemical characterization

#### Hydrogeochemical facies, water types of surface water and groundwater

**Fig. 3:**
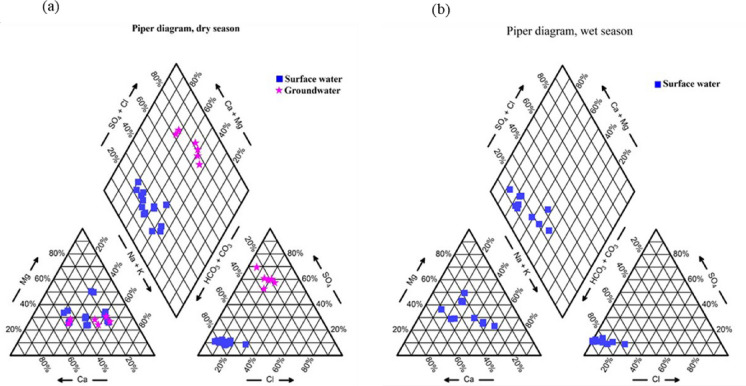
Piper diagram for Moatize’s water classification in dry (**a**) and wet (**b**) seasons

From the Piper’s diagram (Fig. 3a–b), the water was classified as follows for the dry season: Field (I) which is the Ca^2+^-Mg^2+^- Cl^−^-SO_4_^2−^ (80%; 5 out of 6 groundwater); Field (II) which is Na^+^-K^+^-Cl^−^-SO_4_^2−^ (20%; 1 out of 6 groundwater); Field (III) which is Na^+^-K^+^-HCO_3_^−^ (20%; 2 out of 12 surface water) and lastly Field (IV) which is Ca^2+^-Mg^2+^-HCO_3_^−^ (80%; 12 out of 14 surface water). For the wet season, 10 surface water samples fell in Field IV, and 1 surface water sample fell in Field III. In the dry season, a majority of the water samples (70%; 14 out of 20) were classified as calcium-magnesium bicarbonate water type. Additionally, 30% (7 out of 20) were identified as mixed water type, and 30% (6 samples) as calcium-magnesium sulfate water type. For the wet season, the majority of samples were classified as calcium-magnesium bicarbonate (80%; 8 out of 10).

#### Water chemistry controlling mechanism 

**Fig. 4:**
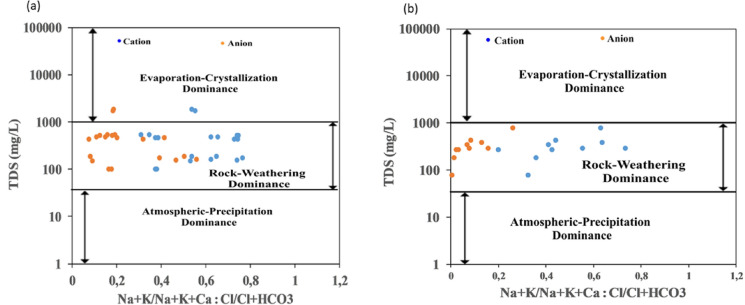
Gibbs plots of water from Moatize in the dry (**a**) and wet (**b**) seasons

The Gibbs’ plot (Fig. 4a–b) showed that 90% of the water chemistry from the dry season was predominantly controlled by rock weathering, while 20% was influenced by evaporation-crystallization (Gibbs, 1970). However, the wet season indicated that 100% of the water is controlled by rock-weathering dominance.

#### Water suitability for irrigation

**Fig. 5:**
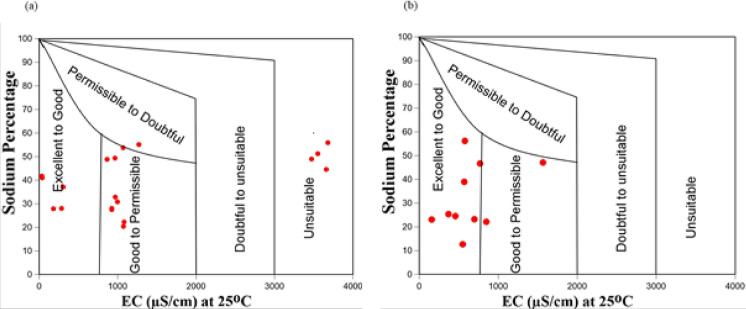
Wilcox classification of water from Moatize in the dry (**a**) and wet season (**b**)

The Wilcox diagram (Fig. 5a–b) indicated that in the dry season, 30% of the water was categorized as excellent-good for irrigation, 20% as unsuitable, 45% as good to permissible, and 5% as permissible to doubtful for irrigation. On the other hand, water from the wet season indicated that 90% of the water was categorized as excellent-good for irrigation, and 10% as good to permissible.

### Spatial distribution maps

**Fig. 6:**
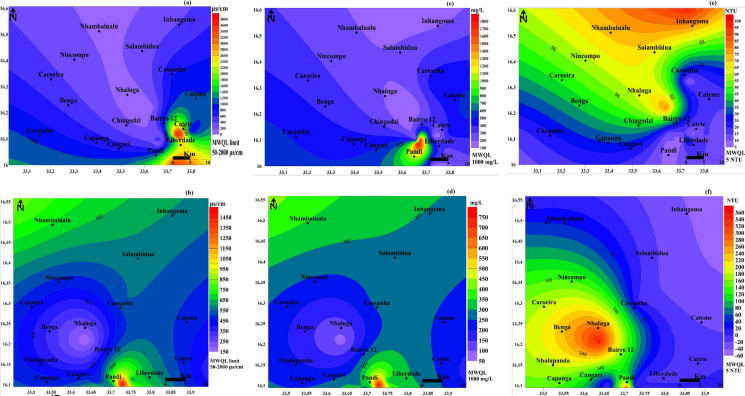
Spatial distribution of key physical parameters in Moatize during the dry and wet seasons: EC (**a**-**b**), TDS (**c**-**d**), and Turbidity (**e**–**f**)

**Fig. 7:**
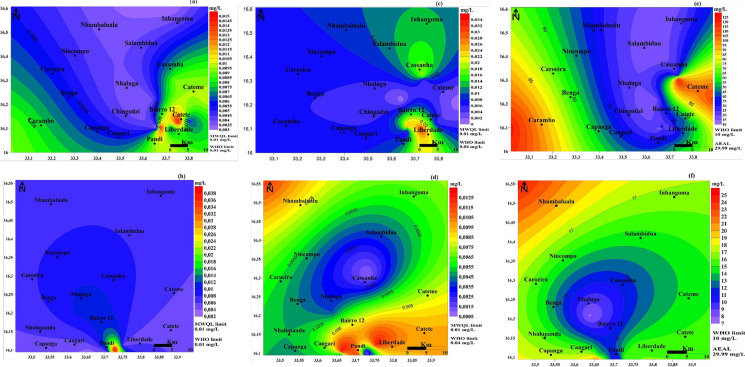
Spatial distribution maps of Pb (**a**-**b**), Se (**c**-**d**), and Si (**e**–**f**) in water from Moatize for the dry and wet seasons

The spatial distribution maps confirm that the sampled water contains both elevated physicochemical parameters and considerable concentrations of PTEs. The geographic spread of physicochemical parameters, specifically EC, TDS, and Turbidity, is detailed in Fig. 6a–f, while the PTEs (Pb, Se, and Si) are shown in Fig. 7a–f. EC concentrations demonstrated a distinct seasonal shift. High EC was consistently determined in the southeastern part of the study area during the dry season (Fig. 6a). These elevated levels migrated to the southern region during the wet season (Fig. 6b). TDS exhibited a similar pattern, with elevated levels found in the southeastern part in the dry season (Fig. 6c) and the southern part in the wet season (Fig. 6d). Furthermore, the highest turbidity concentration shifted from the northeastern part of the region in the dry season to the southeastern part in the wet season.

For the PTEs, high Pb concentrations were located in the southeastern part during the dry season (Fig. 7a), shifting to the southern area in the wet season (Fig. 7b). The Se showed elevated levels in the southeastern region during the dry season (Fig. 7c), but in the wet season, the high levels expanded to cover the southeastern and northwestern parts (Fig. 7d). Si contamination was widespread, showing high concentrations in the western and eastern regions during the dry season, and primarily in the northwestern part during the wet season (Fig. 7e-f).

### Statistical analysis

Based on the classification guidelines developed by Cohen (2013), the correlation analysis of ions, physico-chemical parameters, and PTEs in the water showed distinct patterns between the dry and wet seasons, as detailed in Tables 7 and 8. In the dry season, the correlation analysis of ions and physico-chemical parameters (Table 7) revealed strong positive associations (r ≥ 0.50) for numerous pairs, including Na^+^/Mg^2+^, Na^+^/Ca^2+^, Na^+^/ NO_3_^−^, Na^+^/SO_4_^2−^, Na^+^/EC, K^+^/ Ca^2+^, Na^+^/F^−^, K^+^/TDS, Mg^2+^/ Ca^2+^, Mg^2+^/Cl^−^, Mg^2+^/ NO_3_^−^, Mg^2+^/ SO_4_^2−^, Mg^2+^/ F^−^, Mg^2+^/EC, Mg^2+^/TOC, Ca^2+^/SO_4_^2−^, Ca^2+^/EC, Ca^2+^/TOC, Cl^−^/ NO_3_^−^/ F^−^, Cl^−^/ SO_4_^2−^, Cl^−^/TOC, NO_3_^−^/EC, NO_3_^−^/ F^−^, SO_4_^2−^/EC, SO_4_^2−^/TOC, F^−^/EC, EC/TOC. Notably, the pairs Na^+^/Mg^2+^, Na^+^/EC, Mg^2+^/EC, and NO_3_^−^/F^−^showed very strong positive associations, with correlation coefficients of r = 0.94, r = 0.90, r = 0.91, and r = 1.0, respectively, as presented in Table 7. Moderate positive correlations (r = 0.30–0.49) were identified between pairs such as Na^+^/ K^+^, Na^+^/ Cl^−^, Na^+^/TOC, Na^+^/TºC, K^+^/ Mg^2+^, K^+^/ NO_3_^−^, K^+^/ SO_4_^2−^, K^+^/ F^−^, K^+^/TOC, Mg^2+^/HCO_3_^−^, Ca^2+^/ Cl^−^, Ca^2+^/ NO_3_^−^, Ca^2+^/F^−^, Ca^2+^/ HCO_3_^−^, Cl^−^/EC, Cl^−^/Alkalinity, NO_3_^−^/TOC, SO_4_^2−^/hardness, HCO_3_^−^/TOC, F^−^/TOC, TDS/Alkalinity. Weak associations (r = 0.10–0.29) characterized relationships such as Na^+^/ HCO_3_^−^, K^+^/ HCO_3_^−^, K^+^/EC, K^+^/hardness, Ca^2+^/ TºC, NO_3_^−^/TºC, SO_4_^2−^/ F^−^, SO_4_^2−^/Alkalinity, HCO_3_^−^/F^−^, HCO_3_^−^/Alkalinity, HCO_3_^−^/hardness, F^−^/ TºC, F^−^/hardness, TºC/alkalinity, pH/Alkalinity (Table 7). Regarding PTEs and physico-chemical parameters in the dry season (Table 8), strong positive associations (r ≥ 0.50) were observed for Mo/Si, Se/V, and Si/Mn. Moderate positive correlations (r = 0.30–0.49) were identified between Pb/TDS, Se/Si, Se/ TºC, Si/TºC, and Mn/TºC. Weak associations (r = 0.10–0.29) characterized relationships including Mo/pH, Mo/ TºC, Pb/Se, Pb/Si, Pb/Mn, Pb/V, Pb/Alkalinity, Si/V, Mn/Alkalinity, Cu/ TºC, Cu/ Alkalinity, pH/Alkalinity, TºC/Alkalinity (Table 8).

**Table 7:** Matrix Pearson correlation of ions and physico-chemical correlation in water from Moatize

Dry season															
	Na^+^	K^+^	Mg^2+^	Ca^2+^	Cl^−^	NO_3_^−^	SO_4_^2−^	HCO_3_^−^	F^−^	T °C	pH	EC	TDS	Alkalinity	Hardness
Na^+^	1														
K^+^	.46	1													
Mg^2+^	**.94**	.41	1												
Ca^2+^	**.77**	**.61**	**.83**	1											
Cl^−^	.39	.00	**.52**	.46	1										
NO_3_^−^	**.69**	.33	**.55**	.47	− .26	1									
SO_4_^2−^	**.60**	.42	**.66**	**.64**	**.72**	.19	1								
HCO_3_^−^	.26	.10	.34	.34	.26	.25	.39	1							
F^−^	**.70**	.35	**.56**	.48	− .24	**1.00**	.21	.27	1						
TºC	.03	.07	− .05	.13	− .01	.01	.00	− .01	.01	1					
pH	− .74	− .59	− .76	− .91	− .30	− .52	− .40	− .29	− .52	− .15	1				
EC	**.90**	.18	**.91**	**.64**	.44	**.63**	**.67**	.28	**.64**	− .06	− .53	1			
TDS	− .08	**.53**	− .06	.19	− .13	− .22	− .27	− .12	− .22	.06	− .28	− .42	1		
TOC	.43	.35	**.50**	**.58**	**.50**	.33	**.82**	.39	.34	− .03	− .31	**.53**	− .30		
Alkalinity	− .34	− .05	− .14	− .01	.47	− .67	.28	.17	− .66	.20	.21	− .24	.04	1	
Hardness	− .12	.16	− .56	− .18	− .19	− .04	.36	.19	.11	− .59	.09	− .67	− .02	− .20	1

Wet season															
	Na^+^	K^+^	Mg^2+^	Ca^2+^	Cl^−^	NO_3_^−^	SO_4_^2−^	HCO_3_^−^	F^−^	T °C	pH	EC	TDS	Alkalinity	Hardness
Na^+^	1														
K^+^	.29	1													
Mg^2+^	**.54**	.35	1												
Ca^2+^	**.70**	**.67**	**.80**	1											
Cl^−^	**.97**	.33	**.50**	**.63**	1										
NO_3_⁻	-.21	.38	− .45	− .26	− .02	1									
SO_4_^2−^	.19	**.85**	.33	.41	.33	**.57**	1								
HCO_3_⁻	.41	.40	.31	.36	.51	.46	**.51**	1							
F^−^	.32	.03	.45	.35	.23	− .31	− .10	.25	1						
TºC	− .62	− .46	− .38	− .67	− .60	− .03	− .18	− .49	− .39	1					
pH	− .11	− .15	− .38	− .16	− .25	− .37	− .50	− .64	− .13	− .12	1				
EC	**.92**	.48	**.81**	**.89**	**.88**	− .27	.37	.46	.38	− .65	− .25	1			
TDS	**.91**	.48	**.81**	**.89**	**.88**	− .28	.37	.48	.38	− .64	− .24	**1.00**	1		
Alkalinity	− .22	− .16	− .27	− .20	− .30	− .31	− .27	− .54	− .66	.41	**.57**	− .27	− .24	1	
Hardness	**.70**	**.67**	**.80**	**1.00**	**.62**	− .25	.42	.37	.35	− .66	− .16	**.89**	**.88**	− .20	1

For Tables 7 and 8, bolded values indicate strong positive correlations, specifically those with a Pearson correlation coefficient of r ≥ 0.50.

**Table 8:** Matrix Pearson correlation of potential toxic elements and physicochemical correlation in water

Dry season										
Mo	Pb	Se	Si	Mn	V	Cu	pH	TºC	TDS	Alkalinity
1										
.04	1									
.06	.15	1								
**.50**	.17	.41	1							
− .10	.17	.22	**.67**	1						
− .10	.14	**.64**	.10	.05	1					
− .07	− .21	.17	− .20	− .18	− .19	1				
.13	− .73	− .19	− .13	− .17	− .19	.29	1			
.23	.03	.32	.32	.33	.02	.20	− .22	1		
− .04	.44	− .22	− .04	.05	.00	− .22	− .28	.08	1	
.34	.22	− .09	.30	.19	− .25	.12	.21	.10	.04	1

Wet season												
	Mo	Pb	Se	Si	Mn	V	Cu	EC	pH	TºC	TDS	Alkalinity
Mo	1											
Pb	.03	1										
Se	− .75	− .27	1									
Si	− .05	− .56	.19	1								
Mn	− .19	**.53**	− .16	.07	1							
V	− .07	− .71	.31	**.79**	− .16	1						
Cu	− .01	**.51**	− .04	− .69	.14	− .64	1					
EC	.07	− .39	− .26	.41	− .11	.32	− .44	1				
pH	**.52**	.38	− .36	− .33	− .44	− .38	.19	− .25	1			
TºC	.27	.10	− .20	.43	− .07	.22	− .54	− .08	**.55**	1		
TDS	.06	− .41	− .23	.41	− .13	.32	− .46	**1.00**	− .24	− .07	1	
Alkalinity	.07	.06	− .22	− .18	− .27	− .10	.05	− .27	**.57**	.49	− .24	1

In the wet season, the correlation analysis of ions and physico-chemical parameters (Table 8) revealed strong positive associations (r ≥ 0.50) for Na^+^/Mg^2+^, Na^+^/Ca^2+^, Na^+^/ Cl^−^, Na^+^/EC, Na^+^/TDS, Na^+^/hardness, K^+^/ Ca^2+^, K^+^/SO_4_^2−^, K^+^/hardness, Mg^2+^/Ca^2+^, Mg^2+^/ Cl^−^, Mg^2+^/EC, Mg^2+^/TDS, Mg^2+^/hardness, Ca^2+^/Cl^−^, Ca^2+^/EC, Ca^2+^/TDS, Ca^2+^/hardness, Cl^−^/EC, Cl^−^/TDS, Cl^−^/hardness, NO_3_^−^/SO_4_^2−^, SO_4_^2−^/HCO_3_^−^, pH/Alkalinity, EC/TDS, EC/hardness. Notably, the pairs Na^+^/ Cl^−^, Na^+^/EC, Na^+^/TDS, K^+^/SO_4_^2−^, Ca^2+^/EC, Ca^2+^/TDS, Ca^2+^/hardness, Cl^−^/EC, Cl^−^/TDS, TDS/hardness showed very strong positive associations, with correlation coefficients of r = 0.97, r = 0.92, r = 0.91, r = 0.85, r = 0.89, r = 0.89, r = 1.0, r = 1.0, r = 0.89 and r = 0.89, respectively (Table 7). Moderate positive correlations (r = 0.30–0.49) were identified between Na^+^/ HCO_3_^−^, Na^+^/ F^−^, K^+^/Mg^2+^, K^+^/ Cl^−^, K^+^/ NO_3_^−^, K^+^/EC, K^+^/TDS, Mg^2+^/ SO_4_^2−^, Mg^2+^/ NO_3_^−^, Mg^2+^/ HCO_3_^−^, Mg^2+^/ F^−^, Ca^2+^/ SO_4_^2−^, Ca^2+^/ F^−^, Ca^2+^/ HCO_3_^−^, Cl^−^/ SO_4_^2−^, SO_4_^2−^/EC, SO_4_^2−^/TDS, SO_4_^2−^/hardness, HCO_3_^−^/EC, HCO_3_^−^/TDS, HCO_3_^−^/hardness, F^−^/EC, F^−^/TDS, F^−^/hardness, and TºC/Alkalinity. Weak associations (r = 0.10–0.29) characterized the relationships between Na^+^/ K^+^, Cl^−^/ F^−^, HCO_3_^−^/ F^−^ (Table 7). The correlation analysis of PTEs and physico-chemical parameters in the wet season (Table 8) revealed strong positive associations (r ≥ 0.50) for Pb/Mn, Si/V, Pb/Cu, EC/TDS, pH/T, and pH/Alkalinity. Moderate positive correlations (r = 0.30–0.49$) were identified between Mo/Pb, Mo/Alkalinity, Pb/pH, Si/TºC, Si/TDS, V/TºC, and TºC/Alkalinity. Weak associations (r = 0.10–0.29) characterized the relationships between Mo/ TºC, Pb/ TºC, and V/ TºC (Table 8).

### Water quality pollution indices

**Fig. 8:**
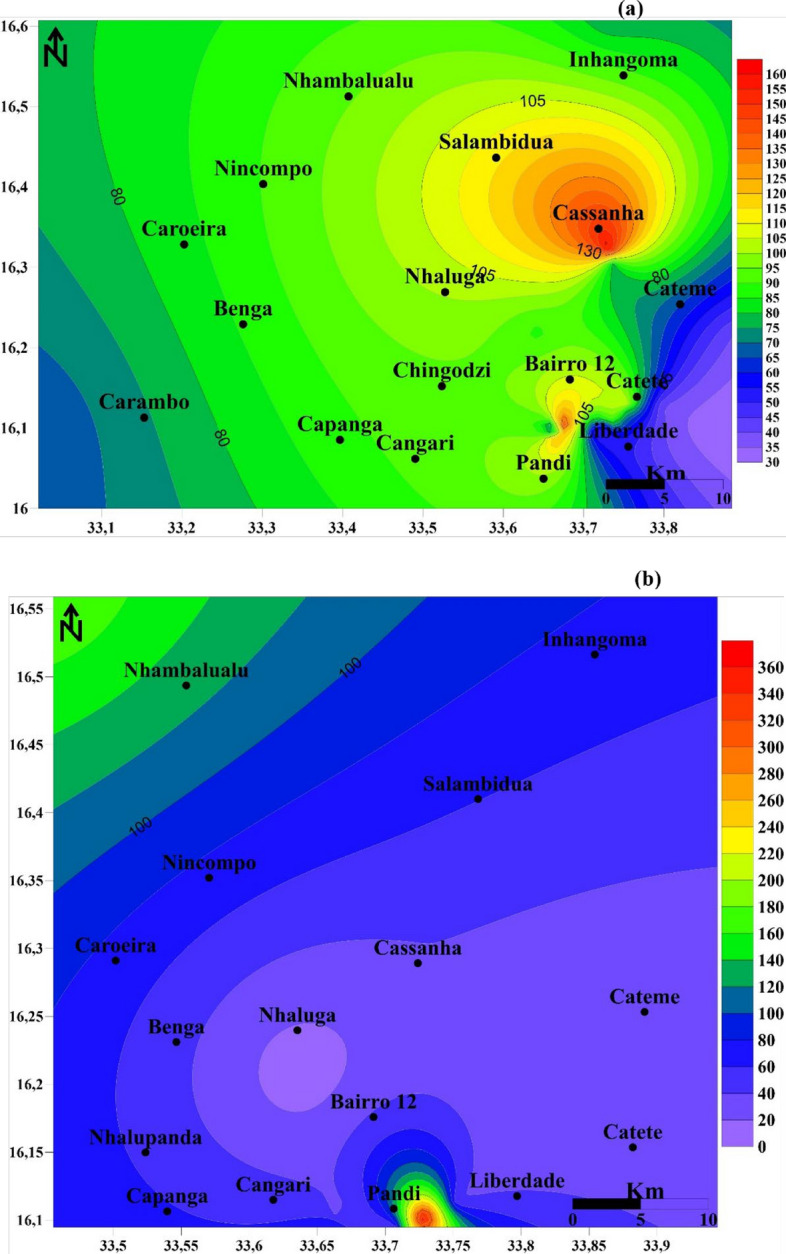
Water Quality Index (WQI) in Moatize across the dry (**a**) and wet (**b**) seasons

Water quality pollution indices for twenty dry-season samples (14 surface, 6 groundwater) and ten wet-season samples are presented in Table S1 and Fig. 8, indicating better water quality during the wet season. The full results of the pollution indices in the Moatize water are presented in Table S2.

During the dry season, all WQI values exceeded 100, indicating that the water was unsuitable for human drinking. Surface water WQI varied by river: Moatize River ranged from 126.54 to 282.91, Revúboè River from 105.7 to 1282.91, Murongozi River from 104.2 to 185.27, and Muarazi River from 123.80 to 185.27. Groundwater WQI showed ranges of 151.74–188.54 in the Moatize River and 120.65–155.61 in the Muarazi River. Conversely, the wet season revealed a much broader spectrum of WQI values, from 29.02 to 4170.59, indicating water quality varied from good to unsuitable for drinking. The water quality of the Moatize River varied significantly, ranging from good conditions to levels deemed undrinkable (Pollution Index: 29.29–3309.87). Both Revúboè River (105.71) and Murongozi River (104.20–158.57) were classified as unsuitable for drinking. Muarazi River water ranged from good to very poor (29.02–97.77), in contrast, the Zambeze River water was critically non-potable, with an exceptionally high WQI of 4170.59.

The Pollution Index (PI) for Moatize’s surface and groundwater ranged from low to highly polluted (0–392.77) (Table S2). In the dry season, the pollution order for surface water was Pb > Se > Mn > Cu. Conversely, the order observed in both wet season surface water and groundwater was Pb > Se > Cu > Mn.

During the dry season, water (surface and ground) in the Moatize region displayed a range of pollution levels from low to high. Surface water quality in Moatize exhibited a wide range: two samples showed low pollution (indices of 0 and 5.3), whereas two other samples were clearly categorized as moderately polluted (indices between 25.88 and 28.9). The Revúboè River’s pollution ranged from moderate (13.58) to high (40.51), mirroring the Murongozi River, which also spanned from moderate (17.0) to high pollution (146.99). The Muarazi River was consistently highly polluted, with indices from 35.29 to 40.42. Water from the Zambeze River showed a spectrum from low pollution (3.19) to high pollution (37.33–53.45). Groundwater in Moatize exhibited a range from low (4.6) to highly polluted (48.36–58.11), and similarly, Muarazi River groundwater was classified as highly polluted (35.29–32.26).

During the wet season, pollution levels generally shifted. Water samples from the Moatize River ranged from low (0) to moderate pollution (28.9). Both the Revúboè and Murongozi rivers displayed moderate pollution, with indices ranging from 19.7 to 22.9. Muarazi River samples showed a broader range, from low (11.02) to high pollution levels (32.26). Additionally, one sample from the Zambezi River indicated high pollution levels (38.18).

### Non-carcinogenic health risk assessment

#### Nitrates

During the dry season, the oral hazard quotient (HQ_oral_) for nitrate was high (above 1) for both adults and children, indicating a significant potential health risk. Children exhibited the highest HQ_oral_ values, ranging from 5.5E−02 to 7.3E + 01, while adult values spanned 2.9E−03 to 3.9E + 01. In contrast, wet season HQ_oral_ values were significantly lower, ranging from 5.5E-03 to 1.4E-01 for children and 2.9E−03 to 7.2E−02 for adults. The dermal hazard quotient (HQ_dermal_) remained below 1 for all age groups in both seasons, as detailed in Table 9, indicating negligible risk associated with dermal exposure.

**Table 9:** Summary of the calculated non-carcinogenic hazard quotients and hazard index for both oral ingestion and dermal exposure in Moatize

Hazard quotient (HQ)																								
HQ_oral_																								
	Adults									Children														
	Dry season			wet season			SN			Dry season						Wet season						SN		
	Min	Max	Mean	Min	Max	Mean	HQ > 1	HQ < 1		Min		Max		Mean		Min		Max		Mean		HQ > 1		HQ < 1
NO_3_	2.9E−02	3.9E + 01	3.8E + 00	2.9E−03	7.2E−02	4.4E−02	2	28	5.5E−02		7.3E + 01		7.9E + 00		5.5E−03		1.4E−01		4.5E−02		2		28	
Mo	2.1E−01	2.4E−01	2.2E−01	7.1E−03	1.1E−01	5.5E−02	0	30	4.0E−01		4.5E−01		4.2E−01		1.3E−02		2.1E−01		5.9E−02		0		30	
Pb	1.0E−02	1.6E−01	7.6E−02	3.1E−02	1.1E−01	1.1E−01	0	30	1.9E−02		3.0E−01		1.4E−01		5.7E−02		2.1E−01		1.2E−01		0		30	
Mn	1.5E−04	3.5E−02	2.3E−02	1.5E−04	7.4E−04	4.9E−04	0	30	2.8E−04		6.6E−02		4.4E−02		2.8E−04		9.3E−04		4.4E−02		0		30	
Se	8.9E−01	2.6E + 01	1.3E + 01	8.9E−01	1.2E + 01	1.3E + 01	25	5	1.7E + 00		6.0E + 01		2.4E + 01		1.7E + 00		2.2E + 01		1.3E + 01		30		0	
V	7.1E−03	3.0E−01	1.7E−01	7.1E−03	1.4E−01	1.6E−01	0	30	1.3E−02		5.6E−01		3.3E−01		1.0E−02		2.7E−01		1.6E−01		0		30	
Cu	0.0E + 00	1.9E−01	6.7E−02	2.7E−02	8.1E + 00	4.8E + 00	2	28	0.0E + 00		3.5E−01		9.7E−02		5.0E−02		1.5E + 01		5.4E + 00		2		28	

HQ_dermal_																
	Adults								Children							
	Dry season			Wet season			SN		Dry season			Wet season			SN	
	Min	Max	Mean	Min	Max	Mean	HQ > 1	HQ < 1	Min	Max	Mean	Min	Max	Mean	HQ > 1	HQ < 1
NO_3_	2.1E–04	2.8E−01	2.7E−02	2.1E−05	5.1E−04	1.7E−04	0	30	7.0E−04	9.4E−01	9.2E−02	3.5E−05	8.7E−04	2.9E−04	0	30
Mo	7.6E−04	8.6E−04	8.0E−04	6.7E−08	1.1E−06	2.9E−07	0	30	2.6E−03	2.9E−03	2.7E−03	2.3E−07	3.6E−06	9.9E−07	0	30
Pb	3.6E−05	5.8E−04	2.7E−04	1.1E−04	4.0E−04	2.2E−04	0	30	1.2E−04	2.0E−03	9.1E−04	3.7E−04	1.3E−03	7.5E−04	0	30
Se	3.2E−03	1.1E−01	4.8E−02	3.2E−03	4.1E−02	2.5E−02	0	30	1.1E−02	3.9E−01	1.6E−01	1.1E−02	1.4E−01	8.4E−02	0	30
Mn	1.3E−04	3.1E−02	2.1E−02	1.3E−04	6.6E−04	4.4E−04	0	30	4.5E−04	1.1E−01	7.0E−02	4.5E−04	2.2E−03	1.5E−03	0	30
V	2.5E−05	1.1E−03	6.2E−04	2.5E−05	5.1E−04	3.1E−04	0	30	8.6E−05	3.6E−03	2.1E−03	8.6E−05	1.7E−03	1.0E−03	0	30
Cu	0.0E + 00	8.9E−04	2.4E−04	9.5E−05	2.9E−02	1.0E−02	0	30	0.0E + 00	3.0E−03	8.1E−04	3.2E−04	9.8E−02	3.5E−02	0	30

Hazard risk index (HI)								
	HQ_oral_				HQ_dermal_			
	Wet		Dry		Wet		Dry	
	children	Adults	Children	Adults	Children	Adults	Children	Adults
NO_3_	0.45	0.24	**143.5**	**76.86**	0.01	0.00	**1.85**	0.55
Mo	0.59	0.29	**1.25**	0.67	0.00	0.00	0.01	0.00
Pb	**1.16**	0.62	**2.82**	**1.51**	0.01	0.00	0.02	0.01
Se	**130.0**	**69.6**	**498.3**	**267.0**	0.84	0.25	**3.21**	0.95
V	**1.63**	0.87	**6.49**	**3.48**	0.01	0.00	0.04	0.01
Cu	**26.95**	**14.44**	**1.63**	0.88	0.17	0.05	0.01	0.00
Mn	0.00	0.00	0.18	0.09	0.00	0.00	0.28	0.08

However, the overall Health Risk Index (HI) for oral exposure exceeded the unity threshold (HI > 1) for both adults and children in the dry season, indicating a cumulative risk from this exposure pathway. Furthermore, the HI for dermal exposure was also greater than 1 for children during the dry season, suggesting a potential combined dermal risk for this group during drier periods (Table 9).

#### Potentially toxic elements (PTEs)

A human health risk assessment was carried out for six PTEs identified in Moatize’s water (Mo, Pb, Se, Mn, V, and Cu). Table 9 displays the results, with hazard index (HI) values exceeding one shown in bold.

For HQ_oral_, Cu hazard quotients were greater than 1 for both adults and children during the wet season. Similarly, Se also presented HQ_oral_ values above 1 for both age groups in both dry and wet seasons. In contrast, the HQ_dermal_ was consistently low, remaining below the unit threshold (HQ < 1) for both adults and children across all seasons (Table 9).

The HI for oral ingestion exhibited distinct patterns. For adults, HI was above 1 for Se and Cu in the dry season, and for Pb, Se, and V in the wet season. For children, the HI for oral exposure exceeded 1 for Pb, Se, V, and Cu in the dry season, and for all elements except Mn in the wet season. Regarding dermal exposure, the HI stayed below 1 for both adults and children across both seasons, with the single exception of Se, which had an HI greater than 1 for children in the dry season (Table 9).

## Discussion

Local coal and ash samples in the study area are rich in PTEs, which present significant environmental concerns. Elevated trace elements in coal pose risks during mining, processing, and combustion. Burning coal can release these elements as atmospheric pollutants or concentrate them in ash (Senior et al., 2020), leading to environmental contamination. Persistent toxic elements such as Pb and Cr can leach from ash into water bodies (USGS, 2015). Furthermore, thin fly ash particles can travel widely, posing risks to ecosystems and human health through inhalation. The mean concentrations of Co, Cr, Sr, and V analyzed in this study exceeded the benchmarks established by Ketris & Yudovich (2009). Conversely, all elements analyzed in ash from Vasconcelos et al. (2014) fell below these same benchmarks. These findings suggest that local coal characteristics or combustion methods may result in increased element concentrations in waste compared to global averages. All metals present a source of contamination in the broader environment and necessitate rigorous management of ash by-products.

The elemental composition of coal critically impacts water quality and human health, primarily through trace element leaching and AMD (Nguegang & Ambushe, 2025). Although coal is predominantly composed of carbon and oxygen derived from ancient plants (Jovanovski et al., 2023), even minor trace elements can significantly contaminate water resources and pose substantial health risks (Finkelman et al., 2019). Elements such as S, Ca, K, Fe, Mg, Na, P, Ti, Cl, and Ba are of particular concern for water quality. These can exist as distinct minerals, be organically bound within the coal, or dissolve as ions in water (Dai et al., 2023). A major issue arises from S and Fe, especially in the form of Pyrite (FeS_2_). When FeS_2_ is exposed to air and water during mining, it oxidizes to create sulfuric acid. This process drives AMD, drastically lowering the water’s pH (Nguegang & Ambushe, 2025). Such acidic conditions then mobilize a range of toxic elements, including Fe, Al, Mn, Cu, Zn, Pb, and As, into water bodies. This contamination inflicts severe ecological damage and simultaneously poses direct human health hazards through contaminated drinking water or trophic transfer (Nguegang & Ambushe, 2025).

The water temperature and pH consistently remained within acceptable seasonal ranges, indicating no thermal pollution and stable, near-neutral to slightly alkaline conditions, a finding consistent with Marove et al. (2022) and WHO (2017) guidelines, indicating no thermal stress or immediate pH-related health concerns. However, turbidity represents a significant and persistent water quality challenge, becoming particularly acute during the dry season due to the concentration of suspended solids resulting from evaporation (Adjovu et al., 2023). Elevated turbidity directly increases the risk of pathogenic microorganism growth by shielding microbes from disinfection (Fahimah et al., 2023). This poses a direct health risk to consumers, potentially leading to waterborne diseases, such as bacterial, viral, and parasitic infections, if the water is consumed without adequate treatment. In groundwater samples, the observed high turbidity may be attributed to the shallow nature of the wells, rendering them susceptible to surface water contamination (Liu et al., 2023). EC and TDS also signal poor water quality with health implications. Dry season EC was notably high, consistent with other studies (Marove et al., 2022). Even with the expected dilution effect of the wet season, all river waters consistently surpassed the WHO (2021) drinking water limits for EC. In direct contrast to Marove et al. (2022), this study found that high dry-season TDS improved in the wet season. However, the persistent high river EC suggests ongoing elevated dissolved ion concentrations (APHA, 2012), which can cause gastrointestinal issues or laxative effects with chronic consumption (WHO, 2017).

Water quality in Moatize exhibits intricate seasonal variability and a distinct hydrochemical separation between surface and groundwater. Although some parameters adhere to established standards, significant public health concerns arise from widespread exceedances of several contaminants. The persistently high ion concentrations in groundwater indicate either natural geological processes, such as mineral dissolution, or the cumulative effect of long-term surface water infiltration (Zhang et al., 2024). This implies that prolonged consumption of polluted water will pose health risks over time.

The comprehensive hydrogeochemical analysis reveals that Moatize’s water system is largely driven by natural geological processes, primarily rock weathering, especially during periods of high rainfall. However, during the dry season, evaporation-crystallization becomes a significant secondary control, concentrating dissolved solids and impacting water suitability for irrigation. The identified water types (especially calcium-magnesium bicarbonate) are consistent with the regional geology.

Data suggests water chemistry variation is distinctly governed by season. In the dry season, the prevalence of very strong and strong positive correlations among major ions and conservative parameters such as EC points to water–rock interaction and evaporation being the dominant processes (Zhang et al., 2020). Specifically, very strong correlations (r ≥ 0.90) among major ions and EC suggest a shared origin and co-concentration resulting from evaporation (Magero et al., 2023). Furthermore, the strong association of Mg^2+^ and Ca^2+^ with TOC is consistent with complexation by organic matter or facultative transport of these cations (Tahraoui et al., 2024). The strong associations among PTEs reflect their common geogenic source and simultaneous co-release during mineral weathering (Konstantinova et al., 2018). In the wet season, the correlations undergo a significant shift: EC, TDS, and hardness become highly correlated with most major cations, often resulting in very strong associations. This pattern suggests that while initial flushing and dissolution occur, the overall chemistry is mainly controlled by the amount of dissolved material entering the system and subsequent dilution by rainfall (Alkhadher et al., 2025). Strong PTE correlations linked to EC/TDS imply contaminants are being mobilized and transported simultaneously via surface runoff and flushing (Panda et al., 2022).

The analysis confirmed a pattern of longitudinal degradation across all rivers, with physical parameters generally increasing downstream. The Moatize River recorded the highest overall EC and TDS concentrations, indicating the highest cumulative pollution. The distribution of ions and metals exhibited a significant seasonal pattern. During the dry season, the high concentrations observed upstream in all rivers strongly suggest PTEs inputs with minimal flow dilution (Naz et al., 2025). Conversely, NO_3_^−^ displayed mixed trends: high downstream in larger rivers (attributed to cumulative inputs) versus high upstream in smaller rivers (suggesting localized, non-mining sources such as agriculture and grazing) (Fang et al., 2025). During the wet season, this trend reversed, with higher concentrations of major ions downstream in the Moatize and Muarazi Rivers. This is attributed to the flushing effect of increased rainfall, which mobilizes accumulated pollutants and sediments settled downstream (Naz et al., 2025). Throughout both seasons, Pb and Se concentrations consistently remained low upstream, supporting the concept of limited mobilization due to slow chemical weathering (Nordstrom, 2011).

The WQI exhibits significant spatial and seasonal variability, with water quality generally improving during the wet season due to the combined effects of pollutant dilution from increased rainfall and enhanced river self-purification capacity. Crucially, Pb and Se consistently dominate as the primary pollutants in both surface and ground water, indicating their widespread presence and substantial environmental impact in the region.

The hazard quotient (HQ) and hazard index (HI) results for nitrates and other PTEs reveal significant health risks associated with groundwater consumption, particularly in the dry season and for children. The primary health concern associated with high nitrate levels in drinking water is methemoglobinemia, commonly known as “blue baby syndrome” in infants (Singh et al., 2025). The significant decrease in HQ_oral_ for nitrates during the wet season is a well-documented phenomenon. This is primarily due to dilution by increased groundwater recharge from rainfall, which flushes and dilutes the accumulated nitrates. During the dry season, lower water tables and reduced dilution can lead to the concentration of contaminants, including nitrates, which often originate from agricultural runoff (fertilizers), septic systems, and sewage (Sridhar et al., 2025). The consistently low HQ_dermal_ values for nitrates across both seasons and age groups (< 1) align with an understanding that nitrate generally is not well absorbed through the skin, making dermal contact a minor exposure pathway compared to ingestion (Egbueri, 2023).

Potentially Toxic Elements assessment identified Cu and Se as PTEs with HQ_oral_ values above 1 for both adults and children, indicating potential health risks from oral ingestion. Similar to nitrates, HQ_dermal_ values remained below 1 for all age groups and seasons, confirming dermal exposure as a less significant route. Cu is an essential trace element, but excessive intake can be harmful. High levels can cause acute gastrointestinal distress (nausea, vomiting, diarrhea) and, in chronic cases, liver and kidney damage (Keshtmand et al., 2025). Selenium is an essential nutrient, but toxic at high concentrations. Chronic oral exposure to excess selenium can cause selenosis, characterized by symptoms such as hair loss, nail brittleness, skin lesions, and neurological effects (Finkelman et al., 2002).

In contrast, HQ_dermal_ generally presented a low risk, with values largely remaining below the threshold for both adults and children in both seasons. This suggests that skin contact with the water is unlikely to cause adverse non-carcinogenic effects. However, a notable exception was observed for children in the dry season, where the cumulative dermal HI could, in some instances, exceed the safe limit, necessitating closer examination of this particular exposure pathway for this vulnerable group.

The HI for oral ingestion was consistently above one for both adults and children in both the dry and wet seasons. This cumulative HI is particularly significant as it suggests that even if individual elements might not pose a risk on their own, the combined effect of these multiple contaminants through drinking water consistently presents a potential non-carcinogenic health risk for both age groups across all periods (USEPA, 2000). This emphasizes the importance of a holistic risk assessment that considers additive effects. While the dermal HI generally remained within safe limits for adults in the dry season and for all age groups in the wet season, the aforementioned exceedance for children in the dry season remains a point of concern.

## Conclusions

This study investigated the seasonal variations of hazardous elements in surface and groundwater around Moatize, Mozambique’s open-pit coal mines, evaluating the overall pollution status. The research found that local coal contains higher levels of several trace elements compared to global averages. These elements are a primary source of PTEs, and mining significantly increases their mobilization into the environment.

This contamination has a direct impact on water quality. Both surface and groundwater show elevated dissolved substances and high ionic content, signaling potential threats to human and ecosystem health. The region’s predominant hydrochemical facies is a Ca–Mg–HCO_3_ type, indicating that interactions with carbonate minerals strongly influence groundwater chemistry. Overall, Moatize’s water sources are generally unsuitable for drinking, necessitating long-term strategies to secure safe water options for the community.

The human health risk assessment highlights significant dangers. Consuming water contaminated with nitrates and various PTEs poses considerable health risks, particularly during the dry season and for children. Elevated hazard quotients raise serious concerns about potential long-term health hazards, including an increased cancer risk. Children are especially vulnerable to chronic health issues resulting from oral ingestion of these contaminants.

The study highlights environmental and human health indicators that are of concern. Relevant authorities must be informed to adopt and implement effective risk management strategies. Immediate action is crucial to implement control measures and effective mitigation strategies for managing water quality in Moatize. Many industries, including mining, agriculture, and urban development, operate in the area, and all industries have a role to play in finding solutions.

## Supplementary Information

Below is the link to the electronic supplementary material.

Supplementary file1 (DOC 68 KB)

## Data Availability

Data used in this study is available on request.
