# Supplementary material for: Assessment of water quality in moatize, mozambique: possible human health risks from coal mining and use
Source: Environ Geochem Health. 2026 Feb 12;48(4):154. doi: 10.1007/s10653-026-03013-1 (PMC12901165; doi:10.1007/s10653-026-03013-1)
Supplement: Supplementary file 1 — Supplementary file1 (DOC 68 KB) [file 10653_2026_3013_MOESM1_ESM.doc]

**Assessment of Water Quality in Moatize, Mozambique: Possible Human Health Risks from Coal Mining and Use**

Micaela Arlete Jose Chapo Cossa^1^, Hassina Mouri^1, *^, Robert B. Finkelman^2^, Vicente Albino Manjate^3^, Kim Dowling^4^

^1^ Department of Geology, Faculty of Science, University of Johannesburg, Johannesburg 2006, South Africa.

^2^ University of Texas at Dallas, Richardson, TX 75080, United States

^3^ National Institute of Mines, Ministry of Mineral Resources and Energy, Maputo – Mozambique

^4^ School of Science, STEM College, RMIT University, Melbourne, VIC 3001, Australia

*Corresponding Author’s E-mail: [hmouri@uj.ac.za](mailto:hmouri@uj.ac.za)

*Tel: +2711 559 4706

Supplementary Information

**Table 9** Computed pollution indices (WQI and PI) of water from Moatize

| **WQI ranges** |  | Dry season | | Wet season | |
| --- | --- | --- | --- | --- | --- |
|  | Quality | n | Source | n | Source |
| 0 - 25 | Excellent | - | - | - | - |
| 26 - 50 | Good | - | - | 3 | river |
| 51 - 75 | Poor | - | - | 2 | river |
| 76 - 100 | Very poor | - | - | 2 | river |
| >100 | Unsuitable | 20 | 14 rivers, 6 well | 3 | river |
|  | | | | | |
| **PI range** | Category | Dry season | | Wet season | |
|  | Quality | n | Source | n | Source |
| <15 | Low | 6 | 4 rivers, 2 well | 2 | river |
| 15-30 | Moderate | 3 | river | 6 | river |
| >30 | High | 11 | 7 rivers, 4 well | 2 | river |

n – number of samples

**Table S2** Overall pollution indices (WQI and PI) of water from Moatize

| Sample Identification | Dry season | | | | Wet season | | | |
| --- | --- | --- | --- | --- | --- | --- | --- | --- |
|  | WQI | Classification | PI | Classification | WQI | Classification | PI | Classification |
| Sw1 | 528.5 | Unsuitable | 28.9 | Medium | 52.0 | Poor | 19.1 | Medium |
| Sw2 | 229.0 | Unsuitable | 25.9 | Medium | 29.3 | Excellent | 26.5 | Medium |
| Sw3 | 123.8 | Unsuitable | 35.3 | High | 43.8 | Excellent | 18.7 | Medium |
| Sw4 | 282.9 | Unsuitable | 13.6 | Low | 79.7 | Poor | 0.1 | Low |
| Sw5 | 104.2 | Unsuitable | 17.0 | Medium | 4170.6 | Unsuitable | 38.2 | High |
| Sw6 | 955.7 | Unsuitable | 37.3 | High | 29.0 | Excellent | 32.2 | High |
| Sw7 | 210.1 | Unsuitable | 392.8 | High | 51.7 | Poor | 22.9 | Medium |
| Sw8 | 126.5 | Unsuitable | 5.3 | Low | 97.8 | Very poor | 11.0 | low |
| Sw9 | 219.5 | Unsuitable | 0.1 | Low | 105.7 | Unsuitable | 19.7 | Medium |
| Sw10 | 185.3 | Unsuitable | 40.4 | High | 3309.9 | Unsuitable | 19.5 | Medium |
| Sw11 | 238.9 | Unsuitable | 40.5 | High |  |  |  |  |
| Sw12 | 158.6 | Unsuitable | 147.0 | High |  |  |  |  |
| Sw13 | 1193.8 | Unsuitable | 53.5 | High |  |  |  |  |
| Sw14 | 249.6 | Unsuitable | 3.2 | Low |  |  |  |  |
| GW1 | 120.7 | Unsuitable | 35.3 | High |  |  |  |  |
| GW2 | 188.5 | Unsuitable | 48.4 | High |  |  |  |  |
| Gw3 | 151.7 | Unsuitable | 38.2 | High |  |  |  |  |
| GW4 | 155.6 | Unsuitable | 32.3 | High |  |  |  |  |
| GW5 | 183.5 | Unsuitable | 58.1 | High |  |  |  |  |
| GW6 | 238.5 | Unsuitable | 4.0 | Low |  |  |  |  |

Sw: surface water; Gw: groundwater
